# Author Correction: SVEP1 is an endogenous ligand for the orphan receptor PEAR1

**DOI:** 10.1038/s41467-023-37005-x

**Published:** 2023-03-17

**Authors:** Jared S. Elenbaas, Upasana Pudupakkam, Katrina J. Ashworth, Chul Joo Kang, Ved Patel, Katherine Santana, In-Hyuk Jung, Paul C. Lee, Kendall H. Burks, Junedh M. Amrute, Robert P. Mecham, Carmen M. Halabi, Arturo Alisio, Jorge Di Paola, Nathan O. Stitziel

**Affiliations:** 1 Division of Cardiology, Department of Medicine, Washington University School of Medicine, Saint Louis, MO 63110 USA; 2 Medical Scientist Training Program, Washington University School of Medicine, Saint Louis, MO 63110 USA; 3 Division of Pediatric Hematology Oncology, Washington University in St. Louis, St. Louis, MO 63110 USA; 4 McDonnell Genome Institute, Washington University School of Medicine, Saint Louis, MO 63108 USA; 5 Department of Cell Biology and Physiology, Washington University School of Medicine, Saint Louis, MO 63110 USA; 6 Department of Pediatrics, Washington University School of Medicine, Saint Louis, MO 63110 USA; 7 Department of Genetics, Washington University School of Medicine, Saint Louis, MO 63110 USA

**Keywords:** Cardiovascular biology, Mechanisms of disease, Platelets, Extracellular signalling molecules

Correction to: *Nature Communications* 10.1038/s41467-023-36486-0, published online 15 February 2023

In this article the author name Arturo Alisio was incorrectly written as Arturo Aliso. The original article has been corrected.

