# Supplementary material for: SVEP1 is an endogenous ligand for the orphan receptor PEAR1
Source: Nat Commun. 2023 Feb 15;14:850. doi: 10.1038/s41467-023-36486-0 (PMC9932102; doi:10.1038/s41467-023-36486-0)
Supplement: Supplementary file 1 — Supplementary Information [file 41467_2023_36486_MOESM1_ESM.pdf]

Supplementary Information

SVEP1 is an endogenous ligand for the orphan receptor PEAR1

Jared S. Elenbaas, Upasana Pudupakkam, Katrina J. Ashworth, Chul Joo Kang, Ved Patel, Katherine Santana, In-Hyuk Jung, Paul C. Lee, Kendall H. Burks, Junedh M. Amrute, Robert P. Mecham, Carmen M. Halabi, Arturo Aliso, Jorge Di Paola, Nathan O. Stitziel

This file includes:

Figure S1. PEAR1 alters plasma levels of SVEP1, continued

Figure S2. SVEP1 and PEAR1 physically interact and colocalize in tissue, continued

Figure S3. SVEP1 activates AKT signaling through PEAR1, continued

Figure S4. The effects of SVEP1 depletion on metabolism in mice

Figure S5. The effects of SVEP1 depletion on vascular function in mice

Figure S6. SVEP1 activates platelets, continued

Table S1. List of cellular reagents

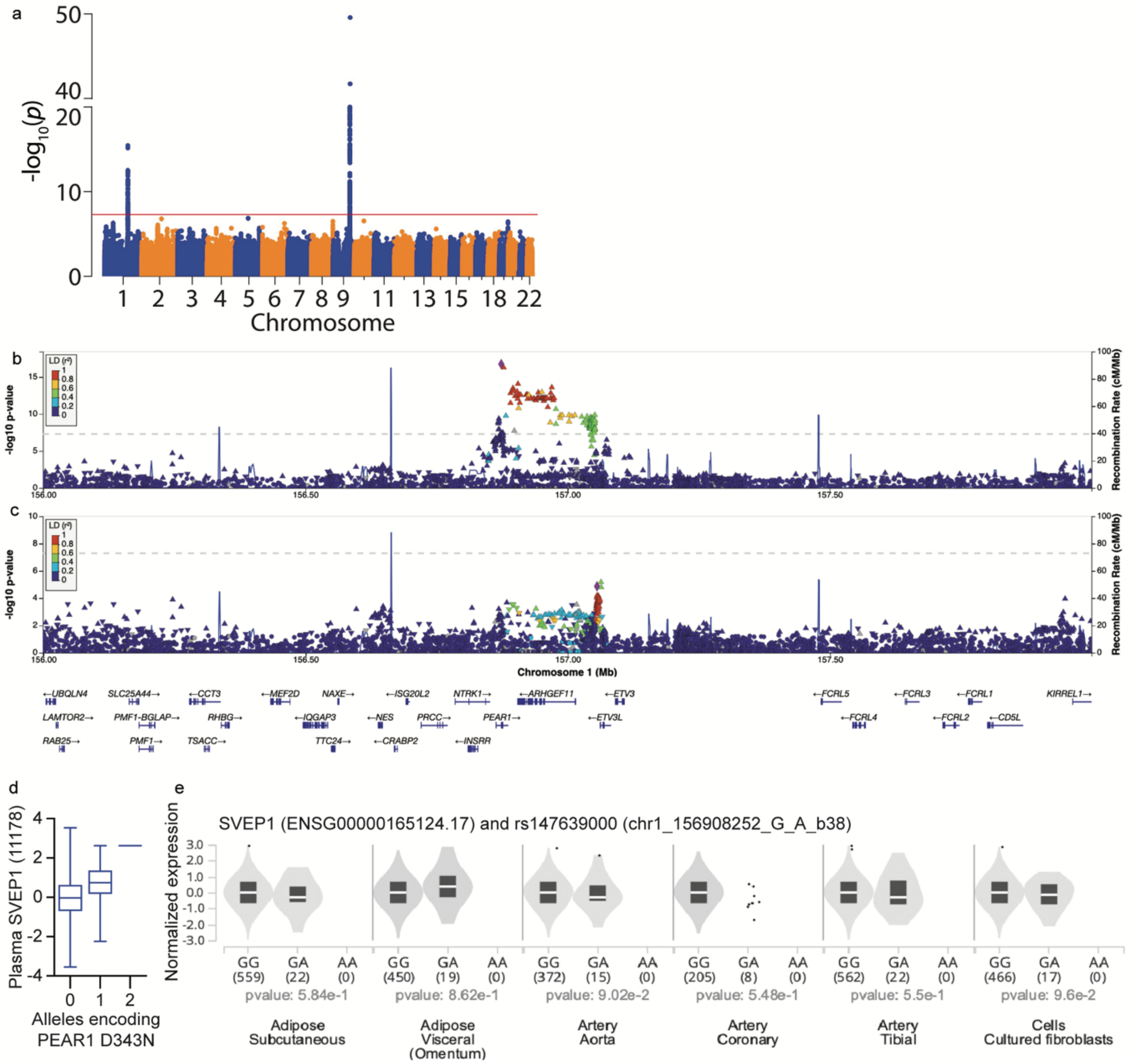

Figure S1. PEAR1 alters plasma levels of SVEP1, continued

(**a**) Manhattan plot showing the unadjusted p-values from an additive model linear regression for autosomal variation with plasma SVEP1 level (aptamer 11109.56.3) in the INTERVAL study. The red line indicates $P \leq 5x10^{-8}$. Modified from Jung and Elenbaas et al., *Science Translational Medicine* 2021 Mar 24;13(586). Reprinted with permission from AAAS.

(**b**) Regional association plot of the results from (**a**) within the PEAR1 locus. Linkage disequilibrium with rs145662369 (denoted by the purple diamond) in the European population is shown. The dashed line indicates $P \leq 5x10^{-8}$. Regional association plots were created by LocusZoom (locuszoom.sph.umich.edu).

(**c**) Regional association plot as in (**b**) after conditioning on rs147639000.

(**d**) Plasma SVEP1 (aptamer 11178.21.3) as a function of allelic copies of rs147639000 (PEAR1 p.D343N) in the INTERVAL study (N=3,301). Beta = 0.70, $P = 1.7x10^{-17}$. Boxes depict upper and lower quartiles with median (center line); whiskers show maximum and minimum values.

(**e**) SVEP1 mRNA levels of selected tissues from GTEx are shown according to number of alternate alleles at rs147639000. Violin plots and statistical significance were directly obtained from the GTEx Project (www.gtexportal.org/home/eqtlDashboardPage). N for each genotype in each tissue are shown below the genotypes.

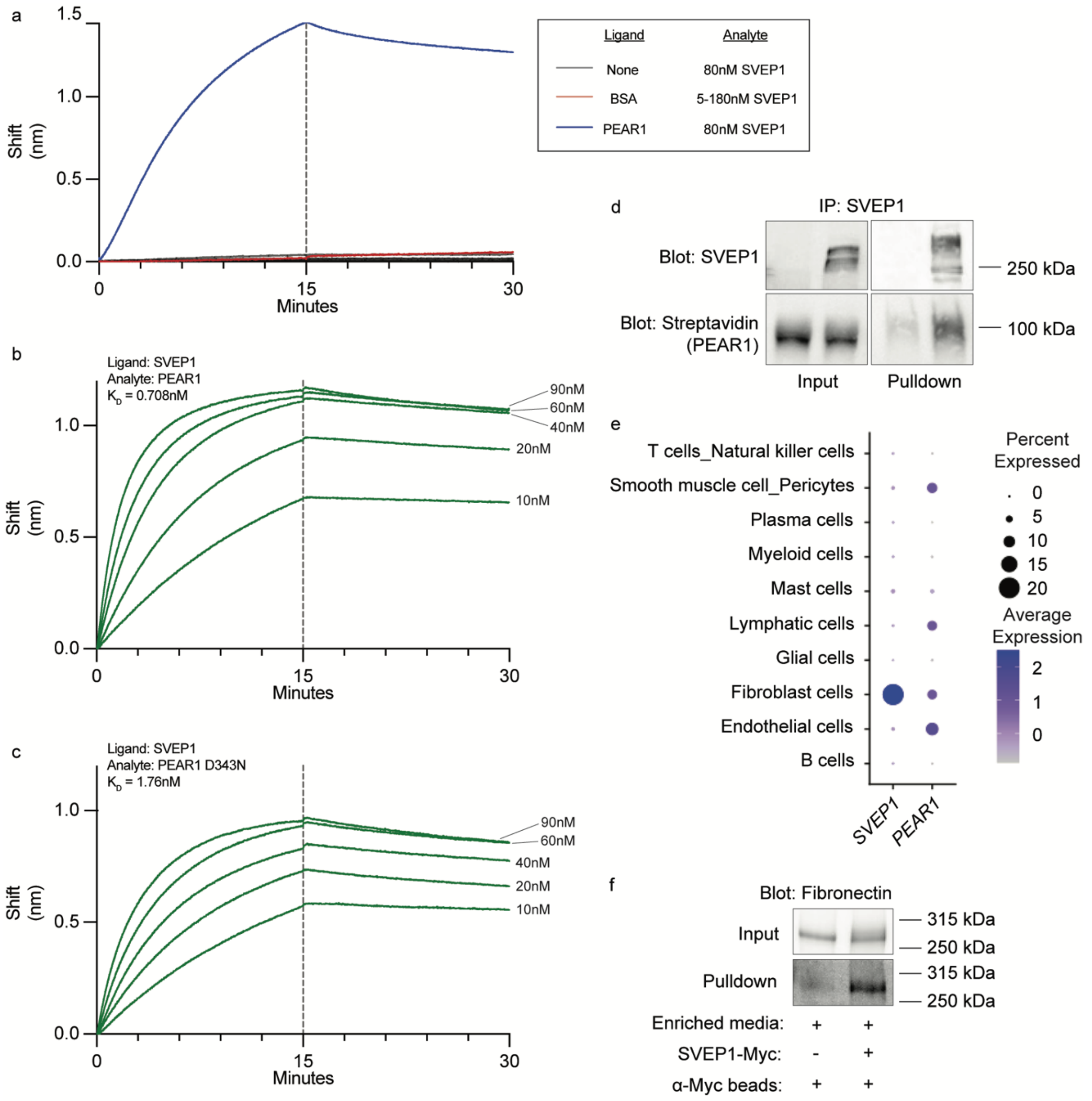

Figure S2. SVEP1 and PEAR1 physically interact and colocalize in tissue, continued
(**a**) Biolayer interferometry control sensorgrams. SVEP1 was analyzed by sensors without a ligand or sensors loaded with biotinylated BSA or PEAR1ECD (representative trace from Figure 2). The dashed line represents the end of the association step and the beginning of the dissociation step.
(**b, c**) Biolayer interferometry sensorgrams. Dilution series of PEAR1 (**b**) or PEAR1 D343N (**c**) were analyzed by sensors loaded with biotinylated SVEP1 (ligand). The dashed line represents the end of the association step and the beginning of the dissociation step.
(**d**) Immunoblots of the indicated proteins after co-immunoprecipitation. Biotinylated PEAR1 was detected using a fluorescent streptavidin probe. The control sample did not contain SVEP1. Additional details listed in Methods.
(**e**) *SVEP1* and *PEAR1* expression in single cell populations of human coronary arteries from publicly available data (reference #42).
(**f**) Immunoblots of Fibronectin from enriched VSMC media after co-immunoprecipitation of SVEP1 with α-Myc beads.

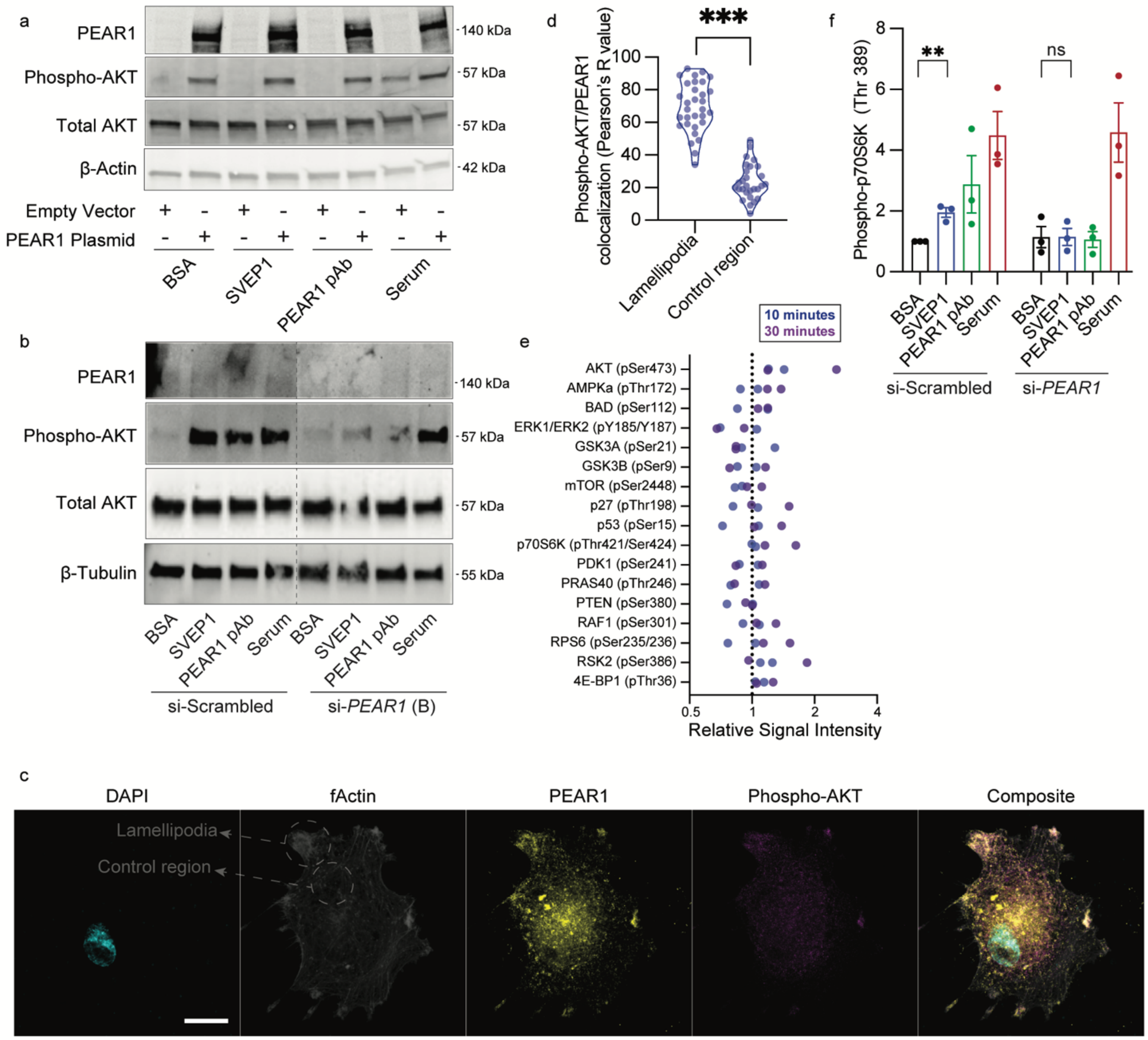

Figure S3. SVEP1 activates AKT signaling through PEAR1, continued

(**a**) 293T cells were transfected with empty vector or a PEAR1-expression plasmid prior to exposure to the listed stimuli. Lysates were analyzed by immunoblot assay.

(**b**) hCASMCs were transfected with scrambled siRNA or anti-*PEAR1* siRNA (construct B) prior to exposure to the listed stimuli. Lysates were analyzed by immunoblot assays for the indicated proteins.

(**c**) Images of HUVECs seeded on immobilized SVEP1 for 60 minutes. Scale bar = 20μm.

(**d**) Quantification of PEAR1 and pAKT colocalization in (**c**), as determined by the Pearson correlation coefficient. Lamellipodia were identified as bundles of fActin on the periphery of cells. Cellular regions not containing lamellipodia were used as control regions. N = 28 and 31, P = 9.6×10^{-19}, unpaired, two-sided t-test.

(**e**) Densitometry quantification of a phospho-protein array for lysates of HUVEC cells exposed for 10 or 30 minutes to immobilized SVEP1 relative to BSA.

(**f**) Densitometry quantification of three independent experiments, represented in Figure 4D. Data are normalized to si-Scrambled, BSA conditions and represent the ratio of phospho-p70S6K (Thr 289) to total p70S6K. P = 0.0038, unpaired, two-sided t-test. Data are presented as mean values +/- SEM.

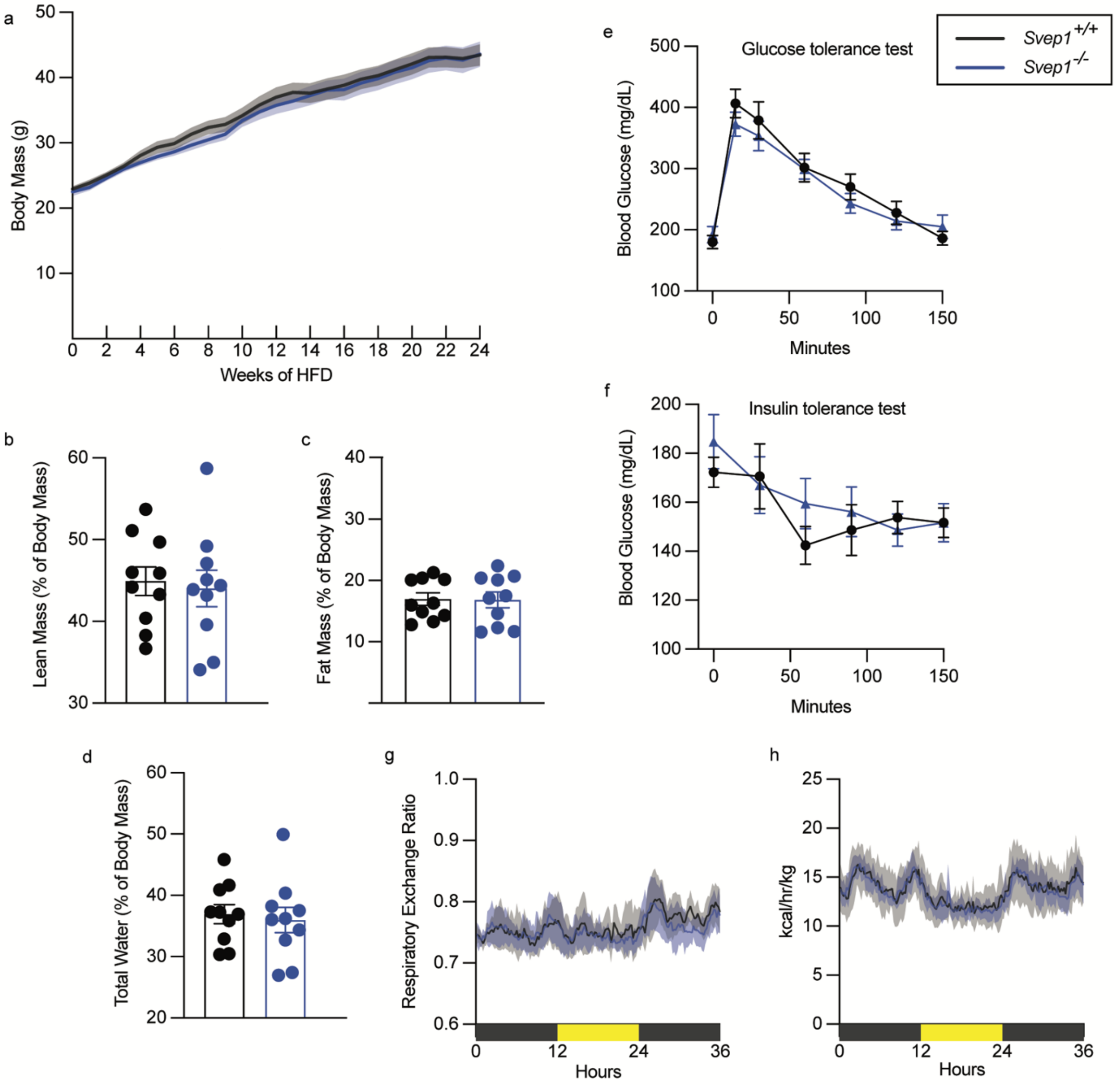

Figure S4. The effects of SVEP1 depletion on metabolism in mice

(**a**) Weekly body mass measurements of *Svep1*$^{+/+}$ and *Svep1*$^{-/-}$ mice after 6 months of HFD feeding. N = 12 and 13 animals. Data are presented as mean values +/- SEM (**a-f**).

(**b-c**) Lean mass (**b**), fat mass (**c**), total water (**d**) of *Svep1*$^{+/+}$ and *Svep1*$^{-/-}$ mice after 6 months of HFD feeding, as determined by EchoMRI^{TM}. N = 10 animals.

(e) Blood glucose measurements of *Svep1*$^{+/+}$ and *Svep1*$^{-/-}$ mice after intraperitoneal glucose administration at 0 minutes. N = 11 and 13 animals.

(**f**) Blood glucose measurements of *Svep1*$^{+/+}$ and *Svep1*$^{-/-}$ mice after intraperitoneal insulin administration at 0 minutes. N = 12 and 13 animals.

(**g**) Respiratory exchange ratio of *Svep1*$^{+/+}$ and *Svep1*$^{-/-}$ mice, as determined by indirect calorimetry. N = 8 animals. Data are presented as mean values (line) and 95% confidence interval (shaded region) (**g**, **h**).

(**h**) Metabolic rate (kcal/hr/kg) of *Svep1*$^{+/+}$ and *Svep1*$^{-/-}$ mice, as determined by indirect calorimetry. N = 8 animals.

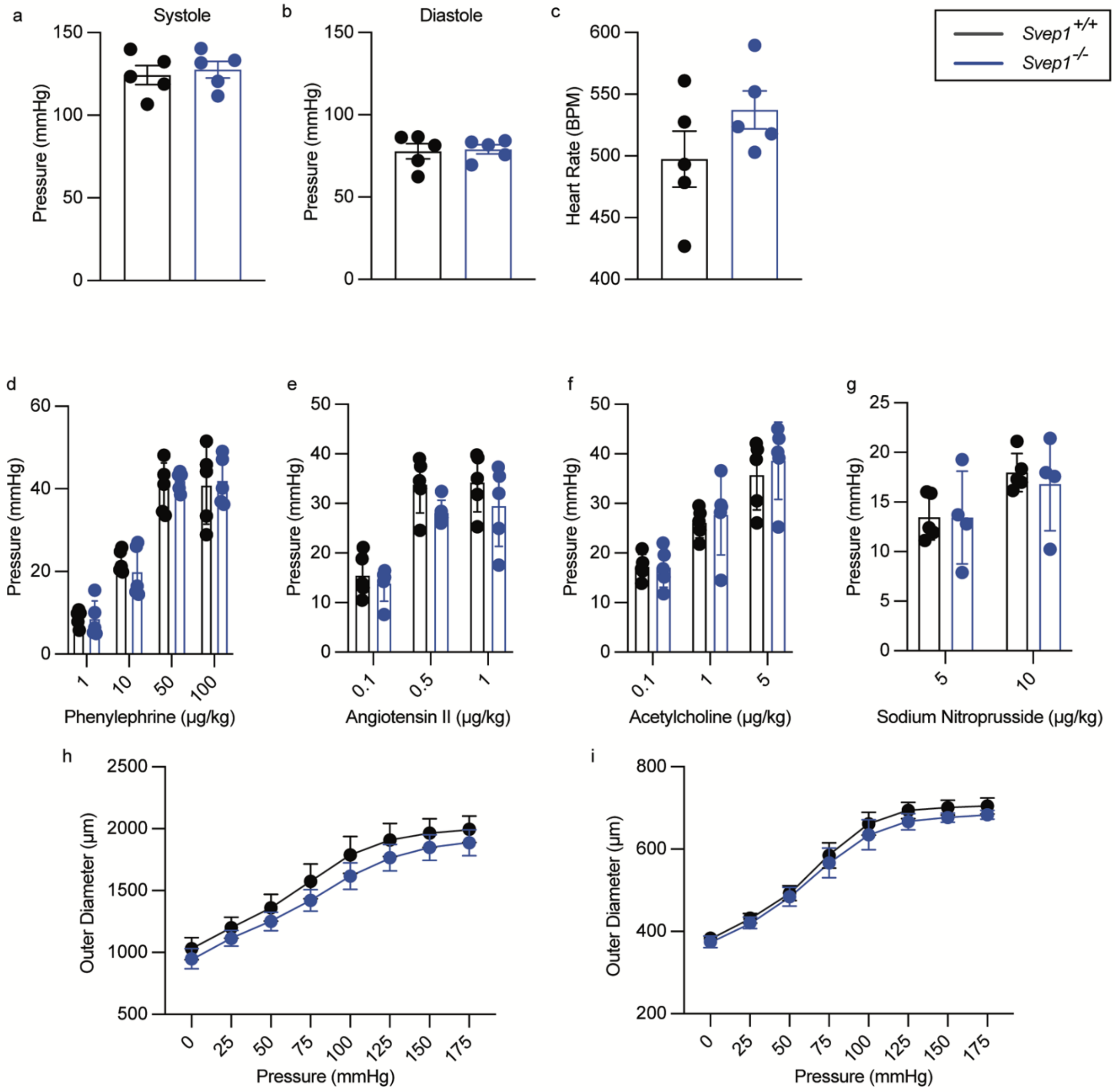

Figure S5. The effects of SVEP1 depletion on vascular function in mice
(**a**) Systolic blood pressure of anesthetized *Svep1*$^{+/+}$ and *Svep1*$^{-/-}$ mice, as determined by arterial catheterization. Data are presented as mean values +/- SEM (**a-i**).
(**b**) Diastolic blood pressure of anesthetized *Svep1*$^{+/+}$ and *Svep1*$^{-/-}$ mice.
(**c**) Heart rate of anesthetized *Svep1*$^{+/+}$ and *Svep1*$^{-/-}$ mice.
(**d**) Change in blood pressure upon venous phenylephrine administration in *Svep1*$^{+/+}$ and *Svep1*$^{-/-}$ mice.
(**e**) Change in blood pressure upon venous angiotensin II administration in *Svep1*$^{+/+}$ and *Svep1*$^{-/-}$ mice.
(**f**) Change in blood pressure upon venous acetylcholine administration in *Svep1*$^{+/+}$ and *Svep1*$^{-/-}$ mice.
(**g**) Change in blood pressure upon venous sodium nitroprusside administration in *Svep1*$^{+/+}$ and *Svep1*$^{-/-}$ mice.
(**h**) Aortic compliance of the ex-vivo aortas from *Svep1*$^{+/+}$ and *Svep1*$^{-/-}$ mice as determined by pressure-diameter tracings.
(**i**) Carotid artery compliance of the ex-vivo carotid arteries from *Svep1*$^{+/+}$ and *Svep1*$^{-/-}$ mice as determined by pressure-diameter tracings. N = 5 animals for all panels except N = 5 and 4 in g.

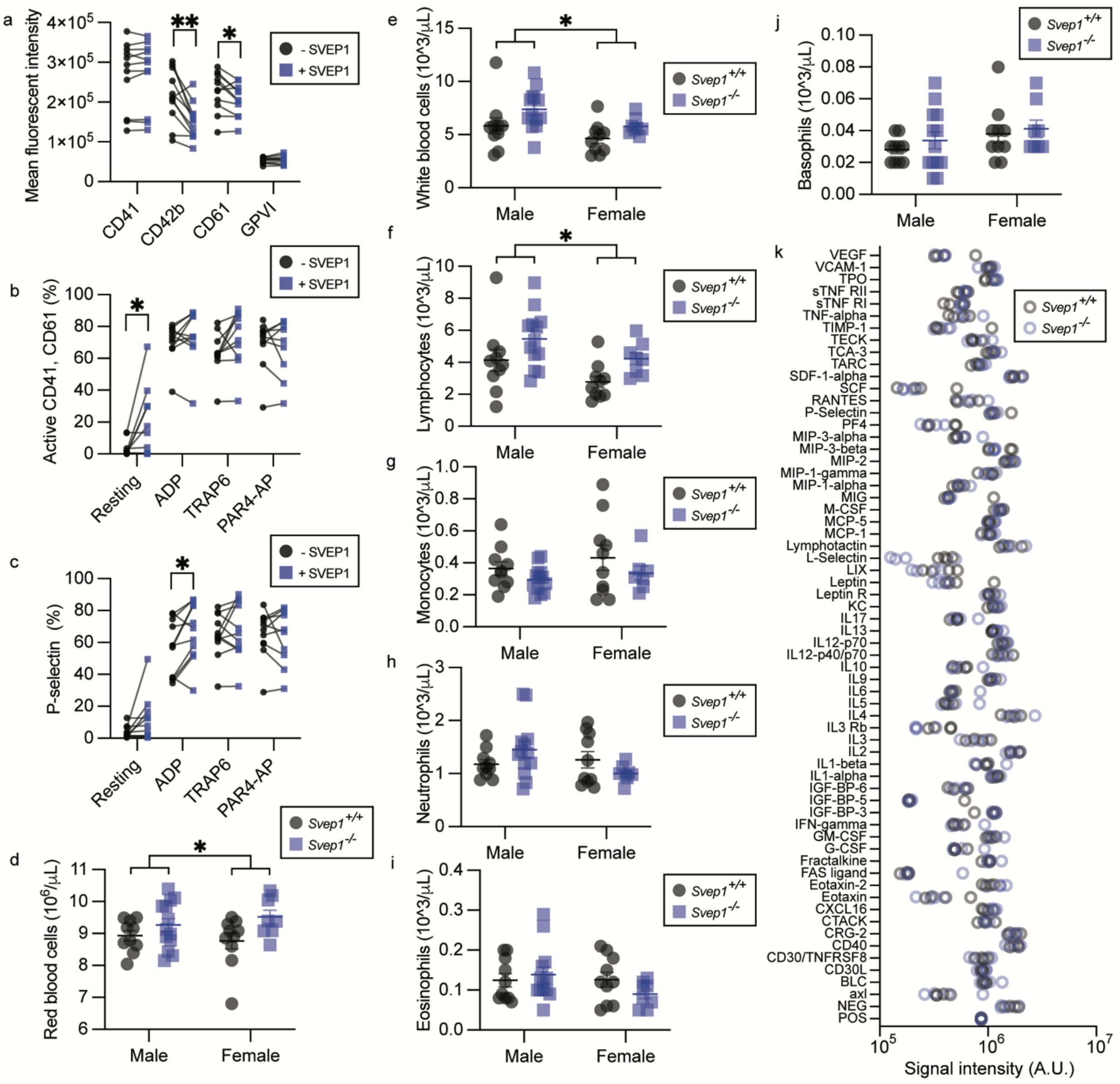

Figure S6. SVEP1 activates platelets, continued

(**a**) Platelet receptor density determined by mean fluorescent intensity (MFI) of the indicated proteins in platelets from whole human blood as determined by flow cytometry. N = 11 independent human samples. Paired t-test. **P = 0.0027, *P = 0.029.

(**b, c**) Activated CD41/61$^+$ (**b**) and P-selectin$^+$ (**c**) platelets were determined by gating after flow cytometry of platelets from whole human blood in resting conditions, or upon stimulation with 10μM ADP, 10μM TRAP6, or 0.1U/mL thrombin. N = 6 and 7 (**b**), and 10 and 11 (**c**) independent human samples. Paired t-test. *P = 0.026 (**b**), *P = 0.019 (**c**).

(**d-j**) RBC count (**d**), white blood cell count (**e**), lymphocyte count (**f**), monocyte count (**g**), neutrophil count (**h**), eosinophil count (**i**), and basophil count (**j**) of blood isolated from *Svep1*$^{+/+}$ and *Svep1*$^{-/-}$ mice as determined by hemocytometry. Two-way ANOVA for all panels, unless otherwise noted. Data are presented as mean values +/- SEM (**d-j**). N = 20 and 21 (**d-f**, **h-j**) or 20 and 20 (**g**) animals. *P = 0.016 (d) *P = 0.021 (e), *P = 0.010 (f).

(**k**) Densitometry quantification of the listed protein using a cytokine array of blood isolated from *Svep1*$^{+/+}$ and *Svep1*$^{-/-}$ mice. N = 4 animals.

Supplemental table of reagents

Reagent Name	Catalogue number	Brand or Company
Fibronectin antibody	ab2413	Abcam Inc.
293T cells	CRL-3216	ATCC
Rhodamine Anti-Tubulin antibody	2004165	Bio-Rad Laboratories, Inc.
Rhodamine Anti-Actin antibody	12004163	Bio-Rad Laboratories, Inc.
PP1	14244	Cayman Chemical Company
Umbilical Vein Endothelial Cells (HUVECs)	50-195-7729	Cell Applications Inc
Total mTOR antibody	2983	Cell Signaling Technology, Inc.
Phospho-mTOR (Ser2448) antibody	5536	Cell Signaling Technology, Inc.
Phospho-p70 S6 Kinase (Thr389) antibody	9234	Cell Signaling Technology, Inc.
Phospho-p70 S6 Kinase (Ser371) antibody	9208	Cell Signaling Technology, Inc.
Phospho-Tyrosine antibody	9411	Cell Signaling Technology, Inc.
Phospho-Akt antibody	4060	Cell Signaling Technology, Inc.
Total AKT antibody	8596	Cell Signaling Technology, Inc.
Total Src antibody	2109	Cell Signaling Technology, Inc.
Phospho-Src antibody	6943	Cell Signaling Technology, Inc.
Myc-Tag antibody	2276	Cell Signaling Technology, Inc.
555 Phalloidin stain (fActin)	50646254	Cytoskeleton, Inc.
Protease and Phosphatase arrest	786-870	G-Biosciences
Mouse IgG isotype contol	31903	Invitrogen
Human primary coronary artery smooth muscle cells (CASMCs)	C-017-5C	Invitrogen
ProLong™ Diamond Antifade Mountant with DAPI	P36962	Invitrogen
IRDye® 680LT Streptavidin	926-68031	LI-COR, Inc.
IRDye® 800CW Streptavidin	925-32230	LI-COR, Inc.
IRDye® 680RD Goat anti-Mouse IgG Secondary Antibody	925-68070	LI-COR, Inc.
IRDye® 800CW Donkey anti-Mouse IgG Secondary Antibody	925-32212	LI-COR, Inc.
IRDye® 680RD Donkey anti-Rabbit IgG Secondary Antibody	926-68073	LI-COR, Inc.
IRDye® 800CW Goat anti-Rabbit IgG Secondary Antibody	926-32211	LI-COR, Inc.
IRDye® 680RD Donkey anti-Goat IgG Secondary Antibody	925-68074	LI-COR, Inc.
IRDye® 800CW Donkey anti-Goat IgG Secondary Antibody	925-32214	LI-COR, Inc.
AF Goat 488	A21467	Life Technologies
AF Rabbit 647	A21443	Life Technologies
Universal nuclease	88701	Pierce
PEAR1 polyclonal antibody	AF4527	R&D Systems
PEAR1 monoclonal antibody	MAB4527	R&D Systems
Human PEAR1 Recombinant Protein	4527PR050	R&D Systems
C-Series Human and Mouse AKT Pathway Phosphorylation Array C1	50-194-8461	Raybiotech Inc.
Mouse cytokine array C3	50-104-3208	Raybiotech Inc.
Dynasore	NC0703465	Selleck Chemical LLC
MK-2206	50-872-5	Selleck Chemical LLC
Rapamycin	73362	StemCell Technologies
Silencer® Select siRNA (si-PEAR1), s51651	4392420	Thermo Fisher Scientific Inc.
Silencer® Select siRNA (si-PEAR1), s51653	4392420	Thermo Fisher Scientific Inc.
Silencer™ Negative Control No. 1 siRNA	AM4611	Thermo Fisher Scientific Inc.
RIPA buffer	AAJ61951AP	Thermo Fisher Scientific Inc.

Table S1. List of cellular reagents
