# Supplementary material for: SVEP1 is an endogenous ligand for the orphan receptor PEAR1
Source: Nat Commun. 2023 Feb 15;14:850. doi: 10.1038/s41467-023-36486-0 (PMC9932102; doi:10.1038/s41467-023-36486-0)
Supplement: Supplementary file 3 — Reporting Summary [file 41467_2023_36486_MOESM3_ESM.pdf]

Corresponding author(s): Nathan Stitziel
Last updated by author(s): Jan 20, 2023

Reporting Summary

Nature Portfolio wishes to improve the reproducibility of the work that we publish. This form provides structure for consistency and transparency in reporting. For further information on Nature Portfolio policies, see our Editorial Policies and the Editorial Policy Checklist.

Statistics

For all statistical analyses, confirm that the following items are present in the figure legend, table legend, main text, or Methods section.

n/a	Confirmed	
☐	☒	The exact sample size (*n*) for each experimental group/condition, given as a discrete number and unit of measurement
☐	☒	A statement on whether measurements were taken from distinct samples or whether the same sample was measured repeatedly
☐	☒	The statistical test(s) used AND whether they are one- or two-sided<br>*Only common tests should be described solely by name; describe more complex techniques in the Methods section.*
☐	☒	A description of all covariates tested
☐	☒	A description of any assumptions or corrections, such as tests of normality and adjustment for multiple comparisons
☐	☒	A full description of the statistical parameters including central tendency (e.g. means) or other basic estimates (e.g. regression coefficient) AND variation (e.g. standard deviation) or associated estimates of uncertainty (e.g. confidence intervals)
☐	☒	For null hypothesis testing, the test statistic (e.g. *F*, *t*, *r*) with confidence intervals, effect sizes, degrees of freedom and *P* value noted<br>*Give P values as exact values whenever suitable.*
☒	☐	For Bayesian analysis, information on the choice of priors and Markov chain Monte Carlo settings
☒	☐	For hierarchical and complex designs, identification of the appropriate level for tests and full reporting of outcomes
☐	☒	Estimates of effect sizes (e.g. Cohen's *d*, Pearson's *r*), indicating how they were calculated

Our web collection on statistics for biologists contains articles on many of the points above.

Software and code

Policy information about availability of computer code

Data collection	No software was used
Data analysis	Tests of statistical significance were conducted in Prism v9.4.1 or R v4.0.3. Mendelian randomization was performed using the R package TwoSampleMR v0.5.6.

For manuscripts utilizing custom algorithms or software that are central to the research but not yet described in published literature, software must be made available to editors and reviewers. We strongly encourage code deposition in a community repository (e.g. GitHub). See the Nature Portfolio guidelines for submitting code & software for further information.

Data

Policy information about availability of data

All manuscripts must include a data availability statement. This statement should provide the following information, where applicable:

- Accession codes, unique identifiers, or web links for publicly available datasets
- A description of any restrictions on data availability
- For clinical datasets or third party data, please ensure that the statement adheres to our policy

Data from the INTERVAL proteomics study were obtained from the European Genome-phenome Archive under Study ID EGAS00001002555. Data from the deCODE proteomics study were obtained from deCODE (https://download.decode.is/form/folder/proteomics). UniProt (ver October 2013) database of mouse proteins was used in the mass spectrometry analysis. GTEx data used for the analyses described in this manuscript were obtained from the GTEx Portal (gtexportal.org) on

10/20/21. The raw mass spectrometry data generated in this study have been deposited in the MassIVE repository under accession number MSV000090134 (doi:10.25345/C54M91F6H). Other data that support the findings of this study are provided in the Source Data file.

Human research participants

Policy information about studies involving human research participants and Sex and Gender in Research.

Reporting on sex and gender	This information was not collected.
Population characteristics	No phenotypic information was collected on the human research participants.
Recruitment	Self-reported healthy controls were recruited in a non-clinical setting without regard to race or sex.
Ethics oversight	Blood collection from consenting healthy controls was conducted in accordance with the Institutional Review Board of Washington University, St Louis.

Note that full information on the approval of the study protocol must also be provided in the manuscript.

Field-specific reporting

Please select the one below that is the best fit for your research. If you are not sure, read the appropriate sections before making your selection.

☒ Life sciences ☐ Behavioural & social sciences ☐ Ecological, evolutionary & environmental sciences

For a reference copy of the document with all sections, see nature.com/documents/nr-reporting-summary-flat.pdf

Life sciences study design

All studies must disclose on these points even when the disclosure is negative.

Sample size	No preliminary data was available to make sample size calculations; therefore, sample sizes were determined a priori based on approximate estimations of biological significance and anticipated variation within a treatment group. Post hoc power analysis determined samples sizes were appropriate.
Data exclusions	Data were excluded prior to analysis whenever a technical error was noted during data collection. Extreme outliers were excluded from the mouse hematological studies using the ROUT method under the most stringent threshold (Q = 0.1%).
Replication	The cell culture and molecular data included in this manuscript are representative of at least two independent experiments which showed similar results. The BLI assay was performed three independent times with similar results. The animal experiments were performed at least once. The protein array experiments were not repeated as they were used as a screening tool; any conclusions from the screening were verified with more sensitive immunoblotting.
Randomization	Excluding the indirect calorimetry measurements, the animal experiments were performed in blinded and randomized fashion. The cellular studies, molecular studies, and the data analysis were performed in unblinded, unrandomized fashion as knowledge of these different conditions was required for sample preparation and analysis.
Blinding	Excluding the indirect calorimetry measurements, the animal experiments were performed in blinded and randomized fashion. The cellular studies, molecular studies, and the data analysis were performed in unblinded fashion as knowledge of these different conditions was required for sample preparation and analysis.

Reporting for specific materials, systems and methods

We require information from authors about some types of materials, experimental systems and methods used in many studies. Here, indicate whether each material, system or method listed is relevant to your study. If you are not sure if a list item applies to your research, read the appropriate section before selecting a response.

Materials & experimental systems

n/a	Involved in the study
☐	☒ Antibodies
☐	☒ Eukaryotic cell lines
☒	☐ Palaeontology and archaeology
☐	☒ Animals and other organisms
☒	☐ Clinical data
☒	☐ Dual use research of concern

Methods

n/a	Involved in the study
☒	☐ ChIP-seq
☒	☐ Flow cytometry
☒	☐ MRI-based neuroimaging

Antibodies

Antibodies used

The following primary antibodies were used: anti-fibronectin (ab2413), rhodamine anti-tubulin antibody (2004165), rhodamine anti-actin antibody (12004163), total mTOR antibody (2983), phospho-mTOR (Ser2448) antibody (5536), phospho-p70 S6 Kinase (Thr389) antibody (9234), phospho-p70 S6 Kinase (Ser371) antibody (9208), phospho-Tyrosine antibody (9411), phospho-AKT antibody (4060), total AKT antibody (8596), total Src antibody (2109), phospho-Src antibody (6943), Myc-Tag antibody (2276), PEAR1 polyclonal antibody (AF4527), PEAR1 monoclonal antibody (MAB4527), CD41-VioBlue antibody (130-105-870), CD61-PE antibody (130-102-628), CD42b-DC649 antibody (M040-3), GPVI-FITC antibody (M011-1), JON/A-PE antibody (M023-2), CD62P-FITC antibody (M130-1), CD41-FITC antibody (303724), CD61-APC antibody (336412), CD42b-PE antibody (303906), GPVI-BV421 antibody (743941), PAC1-FITC antibody (340507), P-selectin-PE antibody (348107).

Antibodies for immunoblot assays were used at 1ug/mL. For immunoflourescent staining, the PEAR1 antibody was used at a concentration of 12ug/mL, and the phospho-AKT antibody was used at a concentration of 15ug/mL. For the PEAR1 pulldown assay, the PEAR1 antibody was used at a concentration of 13.3ug/mL. The antibodies used for flow cytometry of platelets were diluted 1:10 from their stock concentrations.

Validation

All antibodies used in the text are commercially available and have been company validated for the species and techniques used in this manuscript. Pulldown enrichment was verified by immunoblot assays. Signaling assays contained positive and negative controls for additional validation. PEAR1 pAb was further validated in BLI assays.

Eukaryotic cell lines

Policy information about cell lines and Sex and Gender in Research

Cell line source(s)

HUVECs - Cell Applications, Inc
hCASMCs - Invitrogen
293T Cells - ATCC

Authentication

The cells were obtained from companies as listed in the methods. No further validation was performed.

Mycoplasma contamination

Cells were not tested after receiving them from the indicated companies.

Commonly misidentified lines (See ICLAC register)

None were used.

Animals and other research organisms

Policy information about studies involving animals; ARRIVE guidelines recommended for reporting animal research, and Sex and Gender in Research

Laboratory animals

Mus musculus strain C57BL/6, age 6 weeks - 13 months as indicated in the methods section. Svep1 mice with a conditional potential allele were generated by KOMP (Knockout Mouse Project) and crossed with mice expressing the flippase FLP recombinase under the control of the promoter of the human actin beta gene to generate Svep1flx/flx mice. We crossed these mice with Rosa26-CreERT2 (no. 008463, the Jackson Laboratory) mice to generate Svep1flx/+Rosa26-CreERT2 mice. Male and female Svep1flx/+Rosa26-CreERT2 were crossed to generate experimental Svep1flx/flxRosa26-CreERT2 (Svep1-/-) and Svep1+/+Rosa26-CreERT2 (Svep1+/+) littermate control mice. The mice referred in the text as “Pear1-/-” are the Pear1tm1a(KOMP)Wtsi mice generated by KOMP (generously provided by Dr. Bruce Carter, Vanderbilt University). The “Pear1+/+” control mice are age and background matched C57BL/6NCrl mice (Charles River Laboratories) and were acclimated in the same facility as the Pear1-/- mice for at least one week prior to the experiments.

Wild animals

The study did not involve wild animals.

Reporting on sex

Sex was determined by visual inspection of the perineum and external genitalia. The sex of the animal corresponding to certain data points are described in the figures and/or text of the manuscript.

Field-collected samples

The study did not involve samples collected from the field.

Ethics oversight	All animal studies were performed according to procedures and protocols approved by the Animal Studies and Institutional Animal Care and Use Committees of the Washington University School of Medicine.

Note that full information on the approval of the study protocol must also be provided in the manuscript.
